# Stereotactic radiosurgery for brain arteriovenous malformations in patients with hereditary hemorrhagic telangiectasia

**DOI:** 10.1007/s00701-024-05923-4

**Published:** 2024-01-17

**Authors:** Eduardo Orrego González, Sean Runge, Georgios Mantziaris, Natasha Ironside, Jason P. Sheehan

**Affiliations:** Department of Neurological Surgery, University of Virginia Health System, Charlottesville, VA 22908 USA

**Keywords:** Radiosurgery, Telangiectasia, Hereditary hemorrhagic, Intracranial arteriovenous malformation, Intracranial hemorrhages

## Abstract

**Objective:**

Brain arteriovenous malformations (AVMs) in patients with hereditary hemorrhagic telangiectasia (HHT) present different characteristics from sporadic AVMs, and they have lower initial bleeding rates. Conservative management is usually preferred for the treatment of these lesions. In this case study, we present the largest series of HHT patients treated with stereotactic radiosurgery to date.

**Methods:**

We identified eight patients with HHT and 14 AVMs. We retrospectively collected clinical, radiographic, and treatment characteristics of the patients and each AVM.

**Results:**

Most patients in our sample presented with small AVMs. The median volume of these AVMs was 0.22 cm^3^ (IQR 0.08–0.59). Three out of eight patients presented with initial intracerebral hemorrhage (ICH). The majority of lesions had low (12/14) Spetzler-Martin grades (I–II). Median maximum and margin doses used for treatment were 36.2 (IQR 35.25–44.4) and 20 (IQR 18–22.5) Gy, respectively. The overall obliteration rate after SRS was 11/14, and the median time to obliteration across all 11 obliterated AVMs was 35.83 months (IQR, 17–39.99). Neurological status was favorable with all patients having a mRS of 0 or 1 at the last follow-up. Symptomatic radiation-induced changes (RIC) after SRS were low (7.1%), and there were no permanent RIC.

**Conclusions:**

Patients with HHT who present with multiple brain AVMs are generally well served by SRS. Obliteration can be achieved in the majority of HHT patients and with a low complication rate. In the current study, initial hemorrhage rates prior to SRS were noticeable which supports the decision to treat these AVMs. Future studies are needed to better address the role of SRS for HHT patients harboring ruptured and unruptured AVMs.

## Introduction

Brain arteriovenous malformations (AVMs) have an estimated prevalence of 18 per 100,000 adults and variable risks of morbidity (30–50%) and mortality (10–20%). [13] Multiple AVMs are less common accounting for 0.3–3.2% of all AVMs, most of the time associated with hereditary diseases such as hereditary hemorrhagic telangiectasia (HHT). [27] HHT is an uncommon autosomal dominant vascular condition, affecting an estimated 1-in-10,000 people in North America.[13] Diagnosis is established either by detecting pathogenic genetic variants, most commonly in the ENG and ACVRL1 genes, or clinically based on the well-established Curacao criteria.[7, 23] It may result in the appearance of one or more AVMs in affected individuals in areas such as the brain, skin, and lungs.[24] Cerebral AVMs appear in 10–20% of HHT patients, and patients have more often multiple lesions at presentation (42.2%).[12] Morbidity and mortality of patients with HHT can result from hemorrhage and embolic phenomenon from the different AVMs throughout the body, including intracranial AVMs.[9] Radiological characteristics and clinical presentation of AVMs in this population are different from patients without HHT.[2]

Typically, AVMs can be treated via resection, embolization, or radiosurgery, and treatment is based on lesion-specific factors (size, location, eloquence; e.g.) and individual patient characteristics. [20, 36] The use of SRS as a primary treatment for AVMs has been long established; it might also be used as an adjuvant to surgical resection and embolization.[5, 20, 27] Due to the characteristics of these lesions and the low prevalence of AVMs compared to the AVMs present in healthy patients, the treatment of AVMs in HHT patients is not standardized. This is the largest series in the literature reporting the use of SRS as the primary modality of treatment for patients with HHT. Herein, we present our institutional experience and an assessment of clinical and radiological outcomes using SRS and AVMs in the setting of HHT.

## Methods

### Patient population

We performed a retrospective single-center analysis of an AVM database at the University of Virginia during the period of 2013 to 2023. The study was approved by the Institutional Review Board at our center with a waiver of informed consent due to the study’s retrospective nature, following PROCESS guidelines.[1] We included patients who had a diagnosis of HHT fulfilling clinical or genetic criteria, which posteriorly underwent stereotactic radiosurgery treatment for their AVMs.[7] Two hundred seventeen patients who underwent radiosurgery were initially screened for HHT. Five out of these patients had confirmed pathogenic genetic variants of the ENG gene, and three were determined to have met the Curacao criteria for a clinical diagnosis of HHT.[8]

### Data collection

A review of the medical records of these patients was conducted in search of the following information: genetic testing for pathogenic ENG or ACVRL1 gene variants or clinical indicators of HHT including multiple episodes of epistaxis, mucocutaneous telangiectasis, presence of visceral AVMs, or known relatives who carry an HHT diagnosis. General patient information such as demographics, age and symptoms at diagnosis, and comorbidities were collected along with AVM-specific information such as location, size, and prior treatments. Treatment-specific information for each AVM such as prescription volume, maximum and prescription dose, and number of isocenters was collected per treatment session. Outcome data including post-SRS hemorrhage, obliteration status at data collection, time to obliteration, radiation-induced change (RIC) presence, and mortality data were collected.

Radiation-induced changes (RIC) presence and resolution were determined based on the presence of hyperintensity on follow-up T2-weighted or FLAIR cerebral magnetic resonance imaging. We assessed the three following scales for each case: Spetzler-Martin, Virginia radiosurgery scale (VRAS), and radiosurgery-based AVM score (RBAS).[26, 30, 32, 34]

### Radiosurgery treatment protocol

Patients underwent placement of a stereotactic Leksell G-frame in the operating room. During frame placement, they received monitored anesthesia administered by an anesthesiologist. Stereotactic MR imaging was then obtained for treatment planning. Pre- and post-contrast thin-slice (1-mm) axial and coronal MR sequences were obtained. When MR imaging could not be obtained because of medical contraindications (such as the presence of a cardiac pacemaker), a thin-slice stereotactic CT scan was obtained with and without contrast administration. Radiosurgical dose plans were formulated under the direction of a neurosurgeon in conjunction with a medical physicist and radiation oncologist. The Leksell Gamma Unit Model C model (Elekta Instruments, Inc.) was used until 2007 when the Perfexion and later the Icon were used. Elekta’s GammaPlan software was used.

### Outcome definitions and patient follow-up

Clinical and neuroimaging evaluations were performed at 6-month intervals for the first 2 to 3 years after radiosurgery and then yearly. Outcome measures included the rates of AVM obliteration, post-SRS hemorrhage, symptomatic radiation-induced changes (RIC), and favorable patient outcome, defined as AVM obliteration without the occurrence of post-SRS hemorrhage or symptomatic RIC.

AVM obliteration was confirmed on MRI when there was a lack of abnormal flow voids or absence of arteriovenous shunting on cerebral DSA.[22] MRI-documented obliteration cases were included due to the reluctance of patients to undergo repeated DSA procedures; the incidence of DSA-related neurological complications is not inconsequential and brain MRI is highly sensitive in identifying AVM obliteration.[22] Radiation-induced changes (RIC) were defined as T2-weighted or fluid-attenuated inversion recovery perinidal hyperintensities being further classified as asymptomatic or symptomatic. [39] We assessed the mortality of the sample and neurological outcome at the last follow-up by mRS (modified Rankin Scale).

## Results

### Patient statistics

The median age at intracranial AVM presentation across the eight identified patients was 44.5 (IQR, 39.5–60.8) years. The group was evenly split between four male and four female patients (Table 1). Three of these patients (3/8) had a rupture of an AVM on presentation, with the other commonly reported symptoms being neurological in nature such as headache, visual disturbances, altered mental status, and balance changes. One patient presented with recurrent epistaxis. Most patients (5/8) harbored one AVM, with only one patient having four AVMs. Three of the eight patients had no or negative genetic testing, resulting in five patients (5/8) with positive genetic markers for HHT, all of which possessed mutations in the ENG gene (Table 1).

**Table 1:** Summary of characteristics and presentations of HHT patients with brain AVMs

Patient	Age at 1st AVM presentation/Dx (yr)	Age (current) (yr)	Sex	Ethnicity	Total AVMs	Rupture status	Presentation	Variant
1	43	63	F	White	4	None	Headache	None
2	64	66	F	White	1	1 prior hemorrhage	Cerebral hemorrhage, altered mental status	None
3	15	41	M	Hispanic	1	1 prior hemorrhage	Cerebral hemorrhage	None
4	40	42	F	White	3	None	Seizure, worsening memory, worsening balance	ENG
5	34	35	M	White	1	None	Epistaxis	ENG
6	54	60	F	White	1	1 prior hemorrhage	Cerebral hemorrhage, headache	ENG
7	42	47	M	Hispanic	2	None	Photophobia, blurred vision	ENG
8	18	22	M	Hispanic	1	None	Visual disturbances	ENG

### AVM and SRS treatment characteristics

Across the 14 AVMs identified within the patients in the cohort, the most frequent location was in the cerebellum (4/14), followed by the parietal lobe (3/14), the occipital (2/14), frontal (2/14), and temporal lobes (2/14), and finally the basal ganglia/thalamus (1/14) (Table 2). AVMs were noticeably more frequently left-lateralized, with nine out of 14 being on the left side of the brain. The mean maximum diameters of these 14 AVMs were noted to have been approximately 0.7 cm (IQR, 0.6–1.14 cm) with 12/14 having a Spetzler-Martin grade of I or II. Eleven AVMs were assigned VRAS values of 0 or 1, with only one AVM being assigned the highest value of 4. The median RBAS was calculated to be 1.25 (IQR, 1.32–1.1) with volumes ranging from 0.08 to 13.74 cm^3^. Venous drainage was mostly superficial in our sample (11/14). Twelve AVMs were treated on a single SRS procedure, while two of the AVMs in this cohort underwent repeat SRS. Median maximum and margin doses were 36.2 Gy (IQR, 35.3–44.4) and 20 Gy (IQR, 18–22.5), respectively (Table 2).

**Table 2:** Characteristics of all AVMs and treatment

Patient	AVM	Location	Associated aneurysm	VRAS	Spetzler-Martin grade	Max diameter (cm)	Eloquence	Deep venous drainage status	Max dose (Gy)	Margin dose (Gy)	SRS volume (cc)	AVM volume(cc)	RBAS
1	1	L Occipital	0	1	2	1	1	Superficial	50	25	0.6	0.72	1.23
Second treatment	1	L Occipital	0	1	2	1	1	Superficial	45.9	23	0.59	0.59	1.26
Third treatment	1	L Occipital	0	1	2	1	1	Superficial	36	18	0.7	0.22	1.34
	2	L Cerebellum	0	1	1	0.7	0	Superficial	46	23	0.59	0.59	1.26
Second treatment	2	L Cerebellum	0	1	1	0.7	0	Superficial	46	23	0.22	0.103	1.27
Third treatment	2	L Cerebellum	0	1	1	0.7	0	Superficial	35.8	18	0.103	0.09	1.33
	3	R Cerebellum	0	1	1	0.6	0	Superficial	50	25	0.222	0.222	1.28
	4	L Frontal	0	1	2	0.5	1	Superficial	36	18	0.116	0.05	1.03
2	1	R Basal Ganglia	0	2	2	0.67	1	Deep	36.4	20	0.405	0.23	1.90
3	1	R Temporal	1	4	4	4.2	1	Deep	32	16	20.9	13.74	2.69
4	1	L Cerebellum	0	0	1	1.3	0	Superficial	40	20	0.641	0.7	1.17
	2	L Occipital	0	1	2	0.5	1	Superficial	20.5	18	0.193	0.014	1.10
	3	L Parietal	0	0	1	0.6	0	Superficial	20.5	18	0.193	0.006	1.10
5	1	L Parietal	0	1	2	0.95	1	Superficial	40	20	0.401	0.351	1.02
6	1	R Cerebellum	0	2	3	1.4	1	Deep	40	20	0.61	0.027	1.38
7	1	L Parietal	1	0	1	0.7	0	Superficial	33.3	20	1.85	0.07	1.15
	2	L Temporal	0	0	1	0.7	0	Superficial	35	21	0.512	0.091	0.85
8	1	R Frontal	0	0	1	2.2	0	Superficial	36	18	2.17	1.1	0.47

### Outcomes

Of the 14 AVMs treated, there was one episode of hemorrhage recorded post-SRS, with ten AVMs being confirmed to have been obliterated via DSA (Table 3). Median time to obliteration across all 11 obliterated AVMs was calculated to be 35.83 (IQR, 17–39.99) months, with follow-up duration lasting on 112.75 (IQR, 48.8–346.68) months **(**Table 3**).** Radiation-induced changes were noted on imaging post-SRS in seven AVMs, with one patient (1/8) having symptoms prior to resolution (headache and ataxic gait). None of the AVMs had permanent RICs at follow-up. All patients had a favorable neurological outcome at the last follow-up with all patients having a mRS score of either 0 or 1.

**Table 3:** Outcomes after stereotactic radiosurgery for treatment of AVMs on HHT patients

Patient	AVM	Post-SRS hemorrhage	Obliteration status	Time to obliteration (months)	RIC	Symptomatic RIC	Mortality	mRS at last follow-up	Follow-up duration (months)
1	1	N	Y	45	Y	N	N	0	531.5
	2	N	Y	82	N	None	N	0	447.83
	3	N	Y	82	N	None	N	0	371.66
	4	N	Y	82	N	None	N	0	271.75
2	1	N	N	None	Y	N	Y	1	430
3	1	N	Y	35.83	Y	N	N	1	99.41
4	1	Y	Y	17.5	N	None	N	1	50.16
	2	N	Y	12.8	N	None	N	1	45.5
	3	N	Y	12.8	N	None	N	1	45.5
5	1	N	N	None	Y	N	N	0	15.16
6	1	N	Y	36	Y	Y	N	1	55.91
7	1	N	Y	17	Y	N	N	0	129.91
	2	N	Y	17	Y	N	N	0	126.08
8	1	N	N	None	N	None	N	0	48.3

*AVM* arteriovenous malformation, *mRS* modified Rankin scale, *N* no, *RIC* radiation-induced changes, *Y* yes

## Discussion

**Fig. 1:**
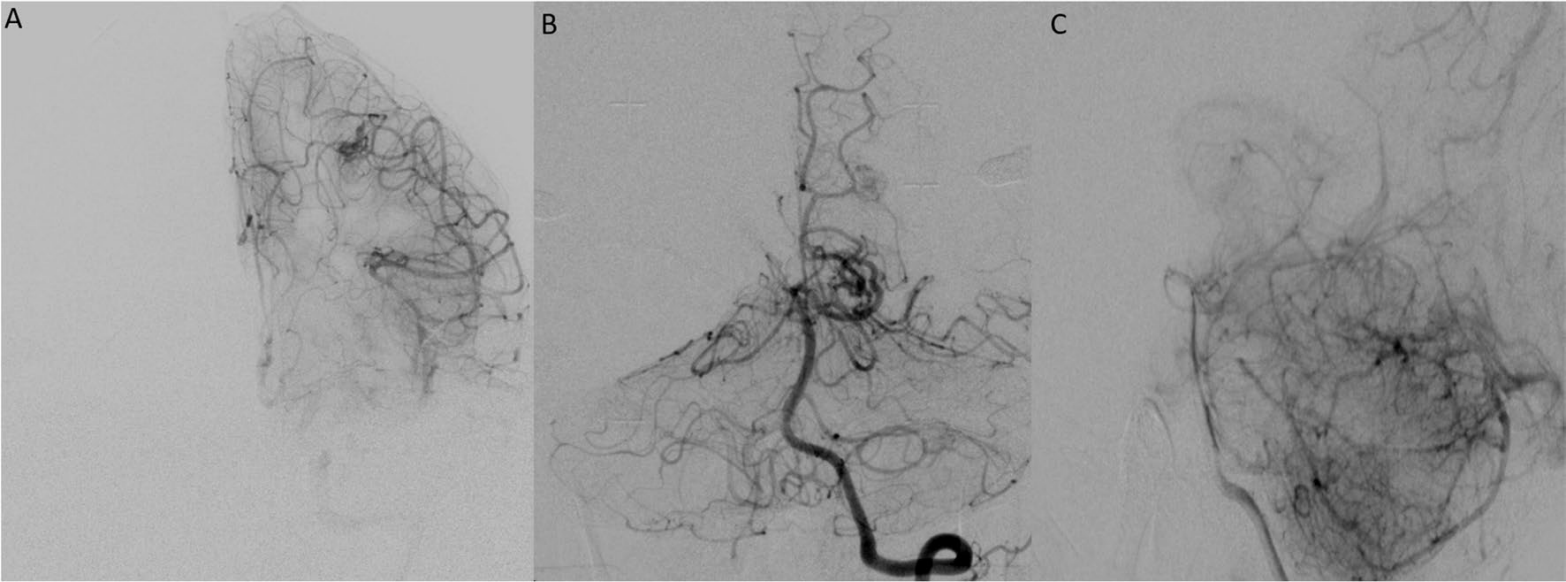
Digital subtraction angiograms of two cases in our sample. Anteroposterior projection of left internal carotid injection in case 8 **(A).** Two AVMs can be seen one in the superficial temporal surface and the second one in the posterior parietal superficial cortex. In case 1 an anteroposterior projection after vertebral injection shows an occipital AVM in the cortical surface **(B)**. The lateral projection of the same patient shows another superficial left cerebellar AVM **(C)**. The sizes of all the lesions were less than 2 cm in maximum diameter

**Fig. 2:**
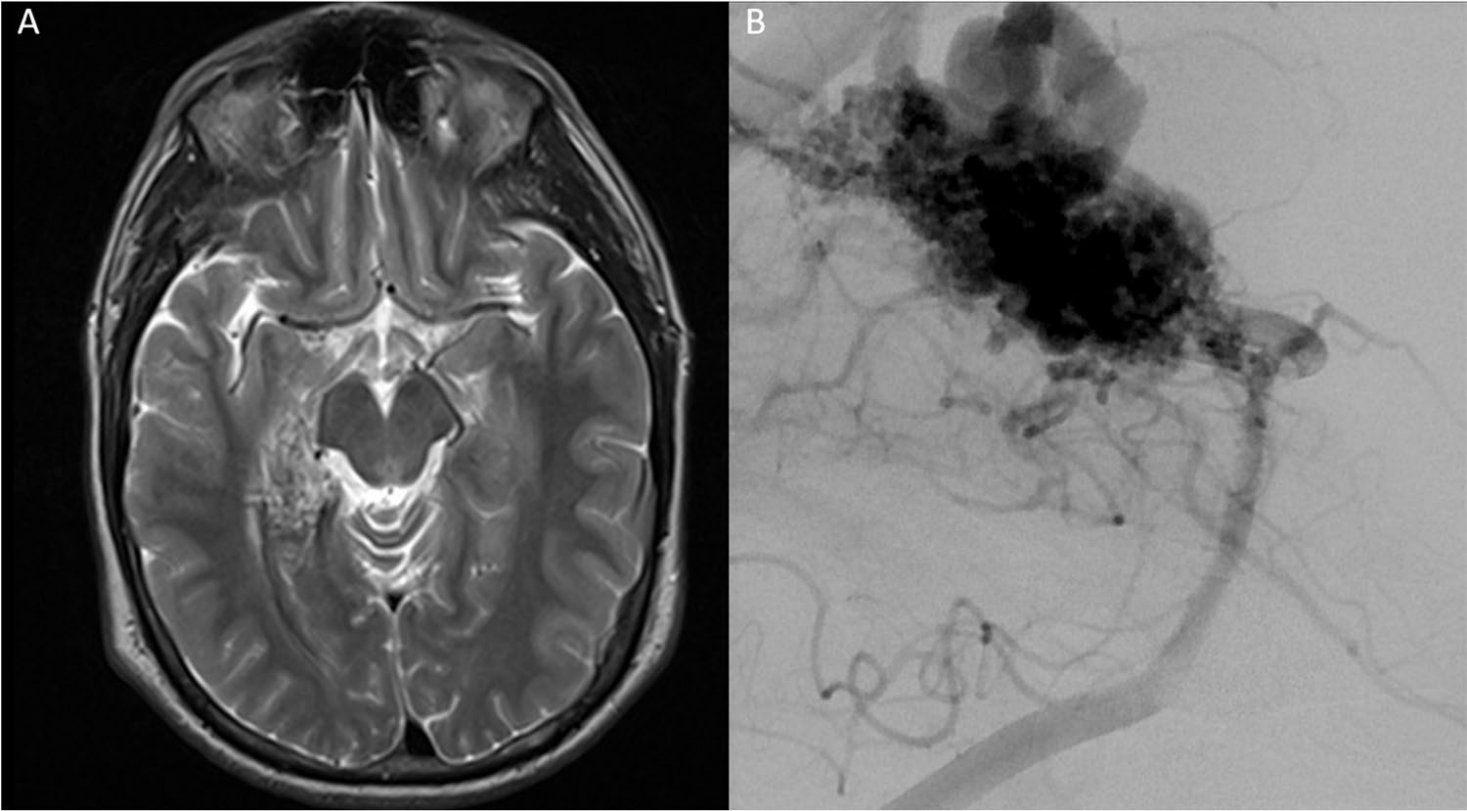
Axial MRI showing an AVM in the right temporal lobe measuring 37.5 mm × 17.4 mm × 32 mm. Lateral view of vertebral injection depicting AVM with deep venous drainage and intranidal aneurysms

Literature on the treatment of AVMs in HHT patients with SRS is limited (Table 4). In this study, we report the baseline characteristics of eight patients with the corresponding vascular characteristics of 14 AVMs and compare them with prior studies.[10, 17, 21, 27, 37] Across a compiled subset of patients with HHT and multiple AVMs treated via SRS the mean age was 28.38 (± 17.55) years, and the sample had an equal distribution between males and females (Table 4). AVMs overall in our study had relatively small volumes, a cortical location, superficial venous drainage, and were multiple in some cases (Figure 1). One patient in our sample presented with a large volume lesion, which is unusual for AVMs in HHT patients (Figure 2). Nineteen out of 32 treated AVMs were assigned a Spetzler-Martin grade of 1 and three were a Spetzler-Marting grade III. This aligns with the observations made in our series, where seven out of 14 AVMs were grade I, and two were grade III. Among 26 AVMs from prior studies, the average maximum diameter was 1.22 cm, similar to the 1.14-cm average maximum diameter observed in this series. These findings place the overwhelming majority of these lesions well within the lowest assignable score for size in the Spetzler-Martin classification. These results are in line with the observed trend that the AVMs in patients with HHT are smaller in size than in patients without inherited diseases. [38]

**Table 4:** Previous reports of SRS for AVMs in HHT patients

Authors & year	Patient #	Type of treatment	Age at 1st AVM presentation/Dx (yr)	Sex	Total AVMs	Rupture status	Presentation	Variant	Location	Spetzler-Martin grade	Max diameter (cm)	Max dose (Gy)	Margin dose (Gy)	SRS volume (cc)	Post-SrS hemorrhage	Time to obliteration (months)	Obliteration status
Kuo et al. 2007 [16]	1	LINAC	12	F	2	Y	Sudden onset headache, photophobia, emesis, dysphasia, visual field loss, new seizures	None	L Frontoparietal, L Occipitoparietal	N/A	0.6, 0.8	23, 21	18.4, 14.7	None	N	6	Y
	2	Resection(1)/LINAC(2/3)	6	F	3	Y	Syncope, hemiparesis	ENG	R Frontoparietal, R Frontal, R Parietal	None	N/A, 0.7, 1.0	20, 20	15, 16	None	N	24	Y
Maarouf et al. 2004 [20]	1	LINAC	26	F	3	N	2 prior CVAs	None	L Temporal x3	I(2), II(1)	N/A	25, 45	20	None	N	18	Y
	2	LINAC	46	M	4	N	Clinical HHT dx based on thoracic AVM/mucocutaneous telangiectasia	None	L Temporal, R Temporal, R Temporal, L Thalamus	II, II, I, II	N/A	25, 25, 34.9, 27.5	20	None	N	24	Y
Ogino et al. 2020 [26]	5	SRS	63	F	4	Y	ICH	None	Right Frontal, Left Frontal, Cerebellum, Brainstem	I, I, II, III	1.2, 1.2, 1.6, 1.2	None	25	0.52, 0.58, 1.33, 0.58	Y	None	Y, N, Y, Y
	6	SRS	43	F	2	N	Headache	None	Right Frontal, Right Temporal	I, II	1.1, 1.7	None	23	1.2, 1.6	N	None	Y
	8	Embolization/SRS	10	M	4	N	Papilledema	None	Right Parietal, Right Frontal, Left Parietal, Cerebellum	I, III, III, I	1.4, 3.9, 4.3, 1.4	None	16	2.0, 7.7, 14.7, 1.9	N	None	Y, N, N, N
	10	Resection/SRS	28	F	2	Y	ICH	None	Right Occipital, Left Frontal	II, I	0.8, 0.8	None	25	0.23, 0.27	N	None	Y, N
Willemse et al. 2000 [37]	11	SRS	30	F	2	None	Asymptomatic	None	Left Frontal x2	I x2	<3.0	None	N/A	None	None	None	None
Gamboa et al. 2018 [9]	13	SRS/resection	16	F	6	Y	Headache	ENG	R Frontal, L Frontal x2, L Occipital, R Parietal x2	I, I, I, I, II, I	0.7, 0.7, 0.9, 0.4, 1.7, 0.7	None	22	None	None	None	None
	23	SRS	51	M	2	Y	Asymptomatic, family history	ENG	R Occipital, L Occipital	I x2	0.4, 0.5	None	20	None	None	None	None
	35	SRS	16	F	2	N	Epistaxis	ENG	L Cerebellum, R Parietal	II x2	1.0, 1.0	None	None	None	None	None	None
	38	SRS	22	F	2	Y	ICH	None	L Parietal, R Cerebellum	I, N/A	N/A	None	None	None	None	None	None

*AVM* arteriovenous malformation, *ICH* intracerebral hemorrhage, *LINAC* linear accelerator, *SRS* stereotactic radiosurgery

Of the 14 AVMs followed in this series, 11 were confirmed to have been obliterated via DSA as of the time of data collection. Of the three patients without confirmation of obliteration, two had follow-ups of over 42 months; one underwent surgical resection of the treated AVM without recent radiographic follow-up, and the second one died of cirrhosis complications. The remaining patent AVM with follow-up under 24 months had a recent SRS treatment and is under observation. Previous literature shows that 18 out of 24 AVMs were successfully obliterated following SRS. [3, 29, 31] As the obliteration rates in our cohort appear to be similar to the ones reported for sporadic AVMs in the literature, we believe that HHT-related AVMs respond well to radiosurgery; this would make SRS with its minimally invasive profile be an appealing option for these patients that would otherwise potentially require multiple resection in their lifetime.[35] The multiplicity of HHT-related AVMs and the ease of the SRS approach can permit repeated and seemingly favorable treatment for these challenging patients.

Three of the eight patients in our sample and seven out of 12 patients from prior studies with HHT patients presented either with an acute hemorrhage or with a history of bleeding related to their AVMs **(**Table 4**)**. This stands in contrast to the generally accepted opinion that AVMs in HHT patients are at lower risk of rupturing. The reported annualized hemorrhagic risk rate for HHT AVMs is 1–1.3%, compared to the hemorrhagic risk rate of 2.1–3% of AVMs in patients without HHT, conferring a 2.5-fold lower risk than sporadic AVMs. [16, 38] There are contradictory findings on the rate of hemorrhages for patients with HHT and AVMs. We found that our sample and previously reported patients with HHT who undergo SRS can have an initial presentation with hemorrhage in a considerable proportion of patients. All four patients identified to have a pathogenic genetic variant in the previous series were associated with HHT, and they were reported to carry those variants in the ENG gene. Similarly, in this study, all reported pathogenic variants were in the ENG gene (Table 4). Across all patients with HHT, variants within the ENG and ACVRL1 genes are the most prevalent, with mutations in the ENG gene (HHT Type 1) carrying nearly tenfold higher prevalence of cerebral AVMs than in the ACVRL1 gene.[15] This again stands in contrast with noted trends in genetic prevalence, with ACVRL1 variants being noted to account for upwards of 70% of HHT cases with known pathogenic genetic variants.[18, 28] It should be considered, however, that our population may possess different rates of pathogenic variants than those reported in population studies derived from other geographic locations.[18, 28]

Most AVMs of patients related to HHT are small, have low SM grades, and hemorrhage rates appear to be overall lower than in the general population. A number of studies have suggested that HHT-related AVMs can spontaneously regress.[4, 6, 19] For this reason, some contend that the benefit-risk ratio of treating these AVMs is low and therefore should be carefully observed instead.[38] However, data compiled by Brinjikji et al. showed that half of the patients with these AVMs are symptomatic, and 20% of the patients will have a hemorrhage-related event.[2] Our findings support these results as seven out of the eight patients in our group had related symptoms to the AVM and three of them had a cerebral hemorrhage. This suggests that patients with HHT benefit from being treated as it can provide symptomatic relief and prevent a detrimental event such as an intracerebral hemorrhage, even if the rate of hemorrhage is lower compared to sporadic AVMs. Morbidity after AVM hemorrhage can be high (53–81%), which supports treatment in this population.[11] The data compiled from our institution and previous studies show that these AVMs can be treated in these groups of patients with different modalities, with good neurological and radiographic outcomes.

Although resection is often deemed to be the preferred treatment for AVMs of small size and low SM grades, SRS is the option in cases where resection is too risky due to location and medical comorbidities.[5] SRS alone achieves high obliteration rates (86%) in these cases,[27] being similar for patients with multiple AVMs at 5 (82.9%) and 7 (82.9%) years, including patients with HHT.[27] Obliteration rates in our cohort (10/14) were similar to those reported for patients with multiple AVMs of different causes. Adverse effects from SRS in our cohort were slightly higher but comparable as well (7/14) to the rates of sporadic AVMs of small and medium size (34.1–40%).[13, 14] None of our patients had permanent RIC, and only one patient suffered from hemorrhage post-treatment.[13, 14] The difference in values might be due to the different characteristics of the AVMs in HHT, the shorter follow-up, and the small sample size of our cohort compared to other studies. All patients in this study preserved their neurological status, which accounts for the safety of SRS in this population comparable to AVMs in patients without genetic disorders.[5] Surgical resection has been used and assessed in this population comparatively with non-treated patients with HHT.[25] Outcomes were favorable in the treated group, although only one patient had multiple lesions.[25] The decision to treat AVMs in HHT patients should be tailored to each specific case. For instance, hemorrhage rates in patients with HHT who present an initial hemorrhage have a higher rate of having new bleeding.[16, 27] Other factors that have been reported to increase the risk of hemorrhage of sporadic AVMs are an infratentorial location, small size, age, deep venous drainage, an associated aneurysm, and deep location.[5, 11, 33] These should be tailored to each specific case in the HHT patients as they are for sporadic AVMs. SRS allows to treatment of multiple AVMs in a single or multiple sessions which makes it preferable over surgery in these particular cases. Future studies should address these factors in this population.

## Limitations

Some limitations of this study include its retrospective nature and the inherent risk of bias. Data were collected in a single center, and the sample size was small. However, our findings add information to the scarce literature on the treatment of AVMs in HHT patients for future research.

## Conclusions

Characteristics of AVMs in patients with HHT include small size, low SM grades, cortical location, and superficial venous drainage. Stereotactic radiosurgery provides a favorable rate of obliteration and a low toxicity rate for HHT AVMs. Although lesions in this population can have lower percentages of hemorrhage, each individual case should be assessed for management, taking into account the individual characteristics of the patient and the vascular characteristics of the AVM.

## Data Availability

The data which supports the conclusions of this study is available from the corresponding author, HCP, upon reasonable request.

## Abbreviations

AVM: Arteriovenous malformations
DSA: Digital Subtraction Angiography
HHT: Hereditary hemorrhagic telangiectasia
MRI: Magnetic Resonance Imaging
RBAS: Radiosurgery-based AVM score
RIC: Radiation-induced changes
SM: Spetzler-Martin
VRAS: Virginia radiosurgery scale (VRAS)
